# Polyploidy and the petal transcriptome of *Gossypium*

**DOI:** 10.1186/1471-2229-14-3

**Published:** 2014-01-06

**Authors:** Aditi Rambani, Justin T Page, Joshua A Udall

**Affiliations:** 1 Plant and Wildlife Science Department, Brigham Young University, Provo, UT 84602, USA

**Keywords:** Cotton, Polyploid, Gene expression, Petal, Expression level dominance, Homoeo-SNP, Gene expression bias, Homoeolog

## Abstract

**Background:**

Genes duplicated by polyploidy (homoeologs) may be differentially expressed in plant tissues. Recent research using DNA microarrays and RNAseq data have described a cacophony of complex expression patterns during development of cotton fibers, petals, and leaves. Because of its highly canalized development, petal tissue has been used as a model tissue for gene expression in cotton. Recent advances in cotton genome annotation and assembly now permit an enhanced analysis of duplicate gene deployment in petals from allopolyploid cotton.

**Results:**

Homoeologous gene expression levels were quantified in diploid and tetraploid flower petals of *Gossypium* using the *Gossypium raimondii* genome sequence as a reference. In the polyploid, most homoeologous genes were expressed at equal levels, though a subset had an expression bias of A_T_ and D_T_ copies. The direction of gene expression bias was conserved in natural and recent polyploids of cotton. Conservation of direction of bias and additional comparisons between the diploids and tetraploids suggested different regulation mechanisms of gene expression. We described three phases in the evolution of cotton genomes that contribute to gene expression in the polyploid nucleus.

**Conclusions:**

Compared to previous studies, a surprising level of expression homeostasis was observed in the expression patterns of polyploid genomes. Conserved expression bias in polyploid petals may have resulted from *cis-*acting modifications that occurred prior to polyploidization. Some duplicated genes were intriguing exceptions to general trends. Mechanisms of gene regulation for these and other genes in the cotton genome warrants further investigation.

## Background

The genus *Gossypium* currently consists of approximately 45 diploid and six polyploid species [1,2]. The six polyploid species of this genus formed between 1–2 million years ago [3,4]. While these polyploid species are currently geographically separated, their monophyletic origin makes this genus an ideal system to study the effects of polyploidization on gene expression. Two polyploid species, *G. hirsutum* and *G. barbadense*, produce spinnable fiber used by the textile industry and represent the majority of world-wide cotton production. An investigation of the effects of polyploidization on gene expression could further our understanding of the basis of superior cotton fiber qualities and fiber yields in tetraploid cotton cultivars.

Polyploidization causes a simultaneous duplication of all nuclear DNA, and some of the genomic consequences of polyploidization can be dramatic [5-7]. One consequence of polyploidization is unequal expression of homoeologous loci. This phenomenon was first described in cotton, for single duplicate gene pairs using Single-Strand Conformation Polymorphism (SSCP) [1,2,8] and genome-wide using custom DNA microarrays [3,4,9]. Subsequent investigations found that expression biases between duplicate genes could be due to growth stage [10-13] or stress [14] and that the inter-genomic biases were reminiscent of monoallelic expression biases (*i.e.* inter-allelic) in diploid *Homo sapiens*[15-17]. Regardless of the growth stage, tissue, or stress, the degree of bias between duplicated gene pairs was distributed across a spectrum of different expression ratios including the 50:50 ratio of most homoeologous gene pairs [12,18,19]. Of the genes with biased expression in petal tissue, approximately 76% of homoeolog expression biases were immediately apparent after genomic merger, while the remaining 24% of homoeolog expression biases had been molded by evolutionary forces over time [20]. The expression level changes that accompanied polyploidization were considered two distinct phases of duplication gene evolution and have been reported in other natural and synthetic allopolyploid species [6,21-26].

Another consequence of polyploidization is expression level dominance. Expression level dominance has been characterized by the abundance of transcript rather than the transcript origin [4]. It was defined by comparing expression levels in *Gossypium* tetraploids to those in related diploids for a given gene. When the tetraploid gene expression level was statistically indistinguishable from one of the diploids, it was assumed that the diploid with the expression level matching that found in the polyploid was dominant. When many genes throughout the genome exhibited expression dominance, the generalized trend was considered expression level dominance of the genome if one of the two genomes was more frequently dominant than the other genome in the tetraploid nucleus. An expression dominance of one of the two genomes was found in leaf [19,27] and petal [18,20] tissue of interspecific hybrids and natural *Gossypium* polyploids. Expression level dominance has also been observed in other polyploid species such as *Coffea*[28], *Spartina*[6] and wheat [7]. Molecular factors contributing to expression level dominance are still unclear, but the *cis-* and *trans-* interactions of the regulatory machinery in the two distinct genomes are one explanation [29]. External factors could also play a role since temperature was shown to influence the magnitude and direction of expression level dominance in *Coffea* species [28]. Differential epigenetic regulation is another possible explanation.

Previously, the transcript contributions of the two co-resident cotton genomes were quantified by custom microarrays [9,10,18] or with RNA-seq and EST assemblies [19]. However, a more accurate assessment of transcriptome composition is possible through RNA-seq technology because gene expression measurement by RNA-seq is not influenced by probe specificity, ascertainment bias of a template sequence, and cross-hybridization [30,31]. Here, we used RNA-seq and the annotated genes of *G. raimondii* to measure gene expression in several polyploid accessions of cotton within its phylogenetic framework.

## Methods

### Plant material

Six accessions were used in our study: *G. arboreum* (2× = 2n = 26, A_2_), *G. raimondii* (2× = 2n = 26, D_5_), *G. tomentosum* (4× = 2n = 52, AD_3_), *G. hirsutum* cv. Acala Maxxa (4× = 2n = 52, AD_1_; referred to as Maxxa), *G. hirsutum* cv. T×2094 (referred to as Tx; 4× = 2n = 52, AD_1_) and a sterile diploid F_1_-hybrid between A_2_ and D_5_ (1× = 1n = 26; F_1_) (Table 1). The diploid F_1_-hybrid was created by a hand pollination between reduced gametes of diploids *G. arboreum* (A_2_) and *G. raimondii* (D_5_), and its somatic cells only contain 13 chromosomes from each extant diploid genome (Table 1).

**Table 1:** List of plant materials used in this study

**Species name**	**Genome designation**	**Accession**	**Ploidy level**	**Location**	**Raw reads**	**Trimmed reads**
*G. arboreum*	A_2_	AKA8401	Diploid	Africa	46,155,539	42,047,506
*G. raimondii*	D_5_	GN33	Diploid	South America	43,715,468	39,974,015
*G. hirsutum*	AD_1_	Maxxa	Tetraploid	Mexico	42,719,425	36,756,492
*G. hirsutum*	AD_1_	T×2094	Tetraploid	Yucatan Peninsula	47,212,060	43,247,980
*G. tomentosum*	AD_3_	WT936	Tetraploid	Hawaii	41,893,620	38,350,345
*G. arboreum X G. raimondii*	A_2_ x D_5_	Unnamed	F_1_-haploid	NA	43,247,980	40,655,468

Petal tissue was collected from plants growing under controlled greenhouse conditions at the Pohl Conservatory, Iowa State University, USA. Tissue was harvested at the time of full petal expansion after dawn but before pollination. Taking one flower from three different plants made three biological replicates for experiments. Harvested tissue was flash frozen in liquid nitrogen and stored at −80°C until RNA and DNA extraction.

### RNA extractions, RNA-Seq libraries and sequencing

RNA samples were extracted from the three replicates using a modified hot borate method [32]. RNA samples were quantified using Ribogreen (Invitrogen Inc., Grand Island, NY) and their quality was evaluated on an Agilent Bioanalyzer (Agilent Technologies, Santa Clara, CA). As described by Illumina, cDNA was sheared by sonication to a 200–400 bp fragment size (Covaris Inc., Woburn, MA). RNA-seq libraries were prepared according to the Illumina TruSeq RNA library prep kit protocol and sequenced on an Illumina HiSeq using v.2 chemistry at the Huntsman Cancer Center, SLC, UT. The sequencing reads are available at the NCBI Sequence Read Archive under Study: SRP028270 and Experiment: SRX328344.

### Data analysis

#### Quality filtering and quantitative assessment of RNA Seq reads

Reads were filtered and trimmed using sickle with a phred quality threshold of 20 (https://github.com/najoshi/sickle). Diploid and tetraploid sequencing reads were individually mapped using GSNAP [33] to the diploid genome reference of *G. raimondii*[34]. Tetraploid reads were categorized in two groups, A_T_ and D_T_, using PolyCat with an index of 24 M homoeo-SNPs identified between several A_1_/A_2_ and D_5_ accessions [35,36] (Table 2). We assessed the transcript abundance for each gene and converted raw read counts to RPKM (reads per kilobase per million mapped reads).

**Table 2:** Number of reads (Millions) that were categorized from reference mapping RNA-seq reads

**Accessions**	**A- Reads**	**D- Reads**	**X Reads**	**N Reads**	**Mapped total**	**Mapped%**
*G. arboreum*	16.5	0.1	0	14	30.8	73.30%
*G. raimondii*	0	17.1	0	16.5	33.9	84.70%
Diploid F_1_-Hybrid	8	8.4	0.1	15.1	32	78.80%
*G. hirsutum* Maxxa	7	6.7	1.2	13.5	28.6	77.70%
*G. hirsutum* Tx2094	8	7.7	1.4	15.7	33.1	76.60%
*G. tomentosum*	7.3	6.9	1.3	14.2	29.8	77.60%
Total	46.8	46.9	4.1	88.9	188.2	78.10%

A- and D-reads were categorized as belonging to the A- and D-genomes. X-reads exhibited a mixed signal, with evidence for both genomes. N-reads did not overlap a known homoeo-SNP and could not be categorized.

#### Petal transcriptome analysis

Universal Probability of expression Codes (UPC) uses a mixed-model approach to quantify the probability of gene expression in a sample [37]. UPC determined which genes were actively expressed in the petal tissue for each accession. Active genes in all the accessions were called ‘commonly expressed genes’ and they were used to generate GO annotations for the petal transcriptome through Blast2GO [38]. BLASTX was performed on the Fulton Super Computer at BYU. Blast2GO visual tools were employed to build pie charts depicting gene ontology. Utilizing GO annotations and Enzyme Codes (EC) the KEGG IDs were assigned to each gene and the transcript abundance was calculated for KEGG pathways [39].

#### Differential expression analysis

EdgeR was used to normalize expression data and perform differential expression analysis [40]. Two factors were used as explanatory variables in model design matrix: ‘accession’ with four levels (diploid F_1_-hybrid, *G. tomentosum*, *G. hirsutum* Tx2094, *G. hirsutum* Maxxa) and ‘genome’ with two levels (A-genome or D-genome). A single, nested interaction design was used to determine genes significantly differentially expressed between accessions. A separate, simple single factor experiment with 8 levels was used to detect genes differentially expressed between two genomes for each accession. EdgeR performs exact test for the negative binomial distribution coefficients to provide p-values and false discovery rates (q-values) for all the genes. Genes with <0.05 FDR were considered differentially expressed.

#### Expression phylogeny

A phylogeny based on expression levels of the genes from all the accessions was built using the neighbor-joining algorithm, with sum of squared differences for all genes between accessions within a distance matrix. We built another phylogeny using the neighbor-joining algorithm based on the difference between the two homoeologous gene expression levels.

#### Expression level dominance analysis

To analyze expression level dominance, every gene of each polyploid accession was separately analyzed and characterized according to the relationships between the RPKM values of the different genomes. Genes without expression in petals as determined by UPC were excluded from analysis. Each gene was categorized after comparison of A_2_ and D_5_ expression to the total expression of the polyploid. A matrix was constructed with the number of genes from the two comparisons and these numbers were used to calculate the total number of genes in each category of expression level dominance.

## Results

### Gene expression in cotton petals

A total of 50 bp, single-ended RNA-seq reads were generated from three replicates of each accession (Table 1). Maxxa and *G. tomentosum* had the most RNA-seq reads (> 40 M each) and diploid D_5_ had the fewest RNA-seq reads (~37 M). Each of these reads was mapped (or aligned) to the 13 large pseudo-molecules of the D-genome reference sequence (v. 2.2.1, [34]) containing an initial set of gene annotations. Not all the reads mapped to the reference genome sequence. Perhaps this is because either the initial draft of the D-genome reference did not have all of the genes annotated, or because transcripts mapped to genomic regions outside of annotated genes, or because some of the genes were not assembled to the 13 large pseudo-molecules. Of the annotated genes in the reference pseudo-molecules, 80% had at least one mapped read from the petal tissue.

Approximately 25% of the mapped reads were assigned to each genome (A_T_ and D_T_). If the mapped reads overlapped a homoeo-SNP position (SNPs between the A- and D-genomes), they were categorized as belonging to one of the two genomes or as a chimeric read because it had A- *and* D-genome nucleotides at different loci in close proximity (A-reads, D-reads, and X-reads, respectively; [35]). If a read did not overlap a homoeo-SNP position, the read was unable to be categorized as originating from either the A_T_- or D_T_-genome (N reads) (Table 2). The number of uncategorized reads was not unexpected given the limited divergence between the A_T_- and D_T_-genome in coding sequences [41].

**Figure 1:**
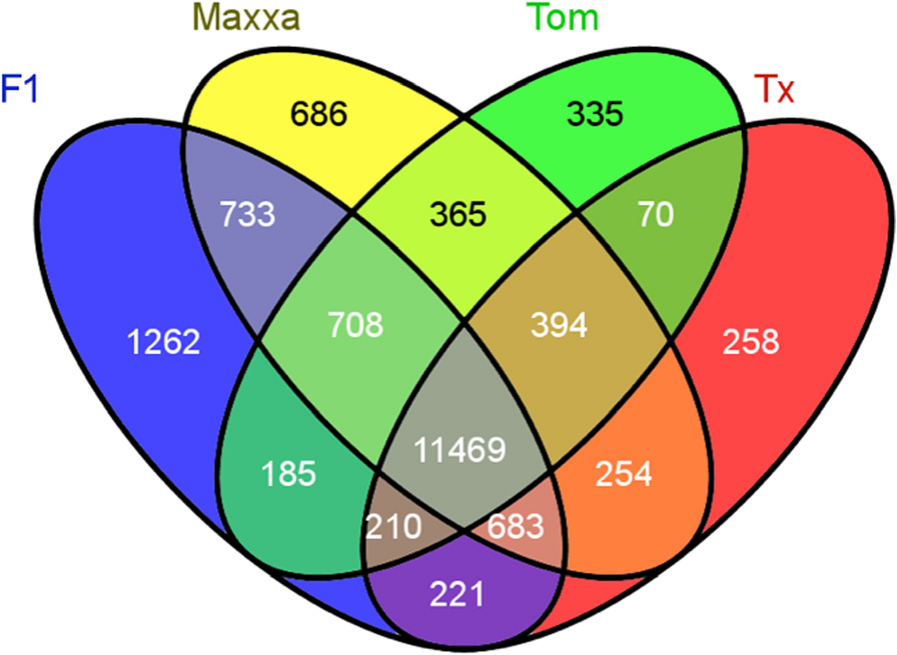
Venn diagram for genes expressed in all the accessions above the background expression level.

Depending on the accession, approximately 45-50% of the genome is expressed in petals based on the UPC analysis. This total is lower than the number of cotton genes found to be expressed in fiber tissue (75-90%) at any developmental stages [12,19]. This could be due to the higher level of canalization of petal tissue compared to fibers. Of 37,223 genes annotated in the reference D-genome, 11,469 genes were commonly expressed in petals of all polyploid accessions as determined by UPC (Figure 1, Table 3). This number of commonly expressed genes represented approximately 80% of the expressed genes in each accession.

**Table 3:** **The number of genes expressed in each *Gossypium *** **accession, the total number shared by every accession, and the number of genes found to have unequal transcript contribution of both genomes (A**_**T **_**and D**_**T**_**) to the transcript pool (genome bias)**

**Accession**	**Total expressed**	**% expressed**	**Total biased**	**% biased**	**A**_**T **_**bias**	**D**_**T **_**bias**	**Bias ratio**
Diploid F_1_-hybrid	15,471	41.6	2,060	13.3	1,027	1,033	0.99
*G. tomentosum*	13,736	36.9	3,706	27.0	1,891	1,815	1.04
*G. hirsutum* Maxxa	15,292	41.0	3,146	20.6	1,556	1,590	0.98
*G. hirsutum* Tx2094	13,559	35.4	2,686	19.8	1,350	1,336	1.01
Commonly expressed	11,469		478*		246*	202*	

*Genes expressed in each of the accessions (Figure 6).

Using Blast2GO, we assigned GO IDs to the common genes based on their RefSeq Blast hits and categorized them into three separate gene ontologies according to their putative function [38] (Additional file 1: Figure S1). The cellular component (CC) ontology had the highest number of assigned GOIDs (88%) followed by the biological process (BP) ontology (17%) and molecular process (MP) ontology (9%). The most abundant GO terms of CC were cytoplasm related (cytoplasm (28%) and cytoplasmic part (27%, Additional file 2: Figure S2). Cellular protein metabolic processes (31%) and kinase activity (41%) were the most plentiful GO terms for the BP and MP ontologies, respectively. Similar distributions among categories have also been reported from the petal tissue of other species, like *Dianthus*[42] and Safflower [43]. Enzyme-coding genes were identified and their role in KEGG enzymatic pathways was determined. A total of 4,565 genes were assigned an enzyme code ID corresponding to 654 different enzymes (*i.e.* many genes were members of large gene families). These 654 enzymes were found to be part of 93 different enzymatic pathways in petal tissue. The enzymatic pathways can be divided into four general categories: Metabolic pathways, Biosynthetic pathways, Degradation pathways and signaling pathways (Additional file 2: Figure S2). Transcript abundance of genes involved in metabolic pathways like starch and amino sugar metabolism was highest in petal tissue compared to other enzymatic pathways. Amongst biosynthetic pathways, biosynthesis of amino acids and flavonoids were most abundant whereas other processes like wax and pigment synthesis had smaller representation.

### Differential gene expression between accessions

**Figure 2:**
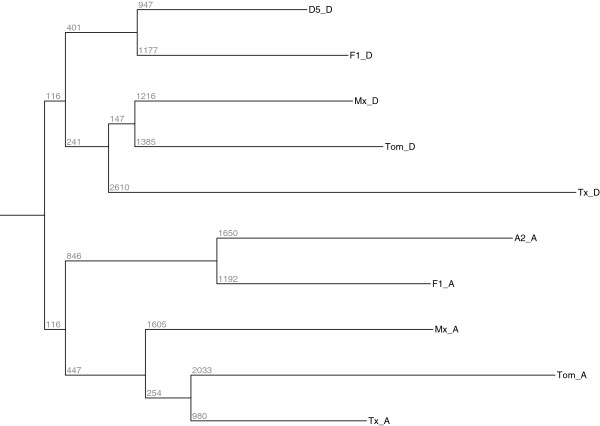
**Phylogenetic tree (neighbor joining in PHYLIP) based on gene expression levels of commonly expressed genes from all the accessions where F1 = diploid F**_**1**_**-hybrid; Mx =** ***G. hirsutum *var Maxxa; Tx =** ***G. hirsutum *var T×2094; Tom =** ***G. tomentosum, _A = A***_***T***_***and _D = D***_***T.***_

Phylogenetic relationships of *Gossypium* species have been well characterized using thousands of genes [41]. It is also possible to ascertain whether variances of gene expression levels follow these evolutionary relationships [18]. Our analysis of *Gossypium* gene expression values resulted in branching patterns similar to that created by previous microarray gene expression levels and to the accepted genetic relationships between species [1,44]. The single expression, phylogenetic tree had two main branches containing the A_T_- and D_T_-genomes, (Figure 2). As expected, the respective genomes of the two *G. hirsutum* accessions, Maxxa and Tx2094, were closely related and clustered together. Using contrasts within EdgeR, differential expression analysis showed that 692 genes were differentially expressed between these two accessions of *G. hirsutum* regardless of the transcript origin*.* There were 1,394 genes differentially expressed between the two accessions of *G. hirsutum* and *G. tomentosum*. The diploid F_1_-hybrid was found to have total expression levels more closely resembling the diploid species than the natural polyploids as might be expected based on its recent origin. The diploid F_1_-hybrid had 2,671 genes differentially expressed between it and the natural polyploids.

### Differential gene expression between homoeologs

The two co-resident genomes of polyploid nuclei did not always contribute equally to the transcript pool. Unequal contribution by the A_T_- and D_T_-genome homoeologs to the cotton transcript pool of any single gene (on the D-genome reference sequence) is referred to as ‘genome bias’ [4,9,18,19,45]. A comparison of the number of biased genes between accessions indicated that the large majority of genes did not have a genome bias (*i.e.* bias ratio of A_T_/D_T_ where A_T_ > D_T_ or *vice versa*) when the genes with 99% UPC expression likelihood were considered. Approximately 20% of genes expressed in petals had a significant bias towards the A_T_- or D_T_-genome (Table 3). Though it wasn’t significantly different, *G. tomentosum* had the highest number of biased genes, followed by Maxxa, then Tx2094, among the natural tetraploids. This ranking was similar to a previous study of petal tissue that also found a higher number of biased genes in *G. tomentosum* than in *G. hirsutum* or the diploid F_1_-hybrid [18]. We included *G. tomentosum* in this study because it had a greater number of homoeologous genes with large expression biases (*i.e*. a broader distribution of A/D expression ratios) than other polyploids. However, we found only subtle differences in the distribution ratios of gene expression among any of the accessions using RNA-seq (Additional file 3: Figure S3). The most notable difference of the distributions was that the diploid F_1_-hybrid had a smaller proportion of expressed genes with a detectable expression bias.

**Figure 3:**
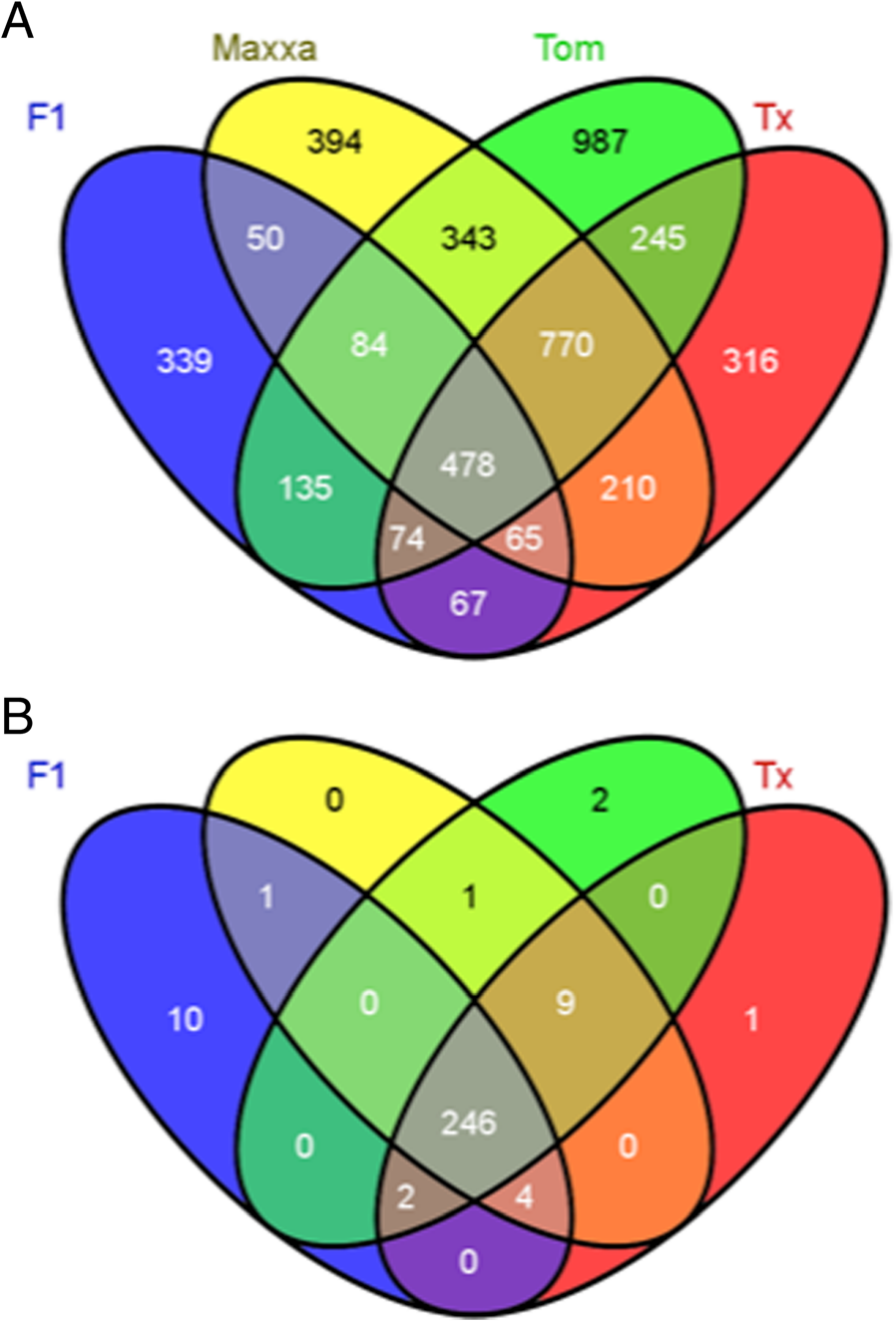
**Venn diagram for number of genes showing homoeologous expression bias in each accession. A)** Total numbers of biased genes in each accession corresponding to the numbers in Table 3. **B)** Of the 478 genes commonly biased in either direction in every accession, only the numbers displayed were biased in the A_T_ direction.

Many commonly expressed genes with homoeologous expression bias were found in more than one accession (Figure 3A). For example, 2,060 genes were identified with an expression bias in the petal tissue of the diploid F_1_-hybrid; of these, 1,292 of these genes were expressed in all accessions. A small set of homoeologous gene pairs (478, ~4% of commonly expressed genes) was found to have an expression bias in each polyploid petal transcriptome. When the degree of bias of these genes was compared to the remaining biased gene pairs for each accession, these 478 biased pairs had a significantly larger degree of bias than the remaining gene pairs (between 1.3 – 1.6 fold greater on average; p < 0.001). For example, the degree of bias for the 478 ‘conserved’ genes was significantly higher than the degree of bias of the remaining 814 genes in the diploid F_1_-hybrid.

The direction of bias for these 478 gene pairs was conserved for most petal transcriptomes with a few exceptions. Of the 478 gene pairs, 246 were consistently biased in the A_T_ direction (Figure 3B) and 202 were consistently biased in the D_T_ direction. These 448 gene pairs were located on all thirteen chromosomes of the D-genome reference. Since we found a significant correlation (p < 0.005) between the number of total genes/chromosome and number of biased genes/chromosome, the homoeologous gene expression bias was probably not related to their chromosomal location. Interestingly, 19 gene pairs in the diploid F_1_-hybrid had consistent, yet contrary biases compared to the natural polyploids (*e.g.* a gene pair within the diploid F_1_-hybrid had an A_T_-bias, but a D_T_-bias was detected in all of the other three natural polyploids). In addition, each of the polyploids had a unique set of a small number of gene pairs with a contrarian directional bias than the remaining accessions (6 genes in *G. tomentosum*, 2 genes in Maxxa, and 1 gene in Tx2094).

Zooming out to reconsider accession totals of biased genes, 339 genes were biased in the diploid F_1_-hybrid but not the natural polyploids, while 770 genes were biased in the natural polyploids but not the hybrid (Figure 3A). The expression bias of these 1,009 genes (339 + 770) was found to be different between the diploid F_1_-hybrid and the natural polyploids. Further examination of the 770 homoeologous gene pairs with conserved expression bias in the natural polyploids found that the direction of expression bias (*i.e.* A_T_ > D_T_ or D_T_ < A_T_) was conserved for all but 1–2 pairs of genes (depending on which two natural polyploids were compared). This conserved direction of homoeologous bias suggested a difference in *cis-*regulation of gene expression between genomes. Inspection of all of the other biases shared between two or more accessions found strong, consistent directions of homoeologous expression bias suggesting that the bias has roots in the independent evolution of the two progenitor species prior to reuniting within the common tetraploid nucleus.

**Figure 4:**
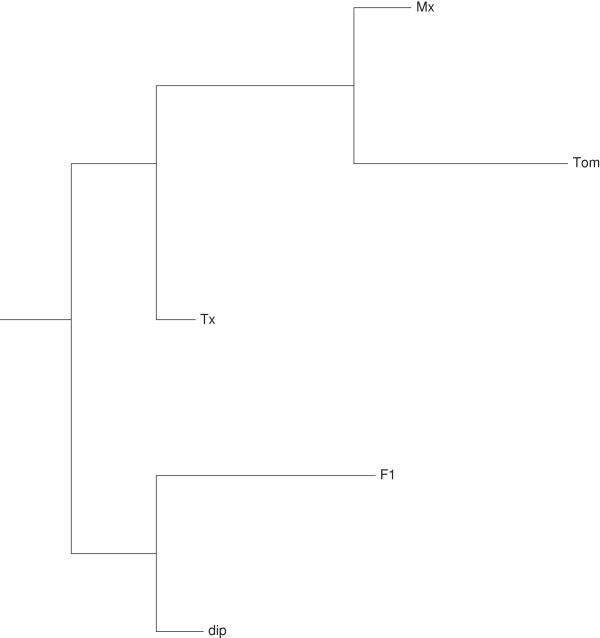
**A phylogenetic tree based on the amount of expression divergence between homoeologous gene pairs (F1 = diploid F**_**1**_**-hybrid; Mx =** ***G. hirsutum *var Maxxa; Tx =** ***G. hirsutum *var TX2094; Tom =** ***G. tomentosum*).**

The degree of bias (*i.e.* expression level differences of A_T_ - D_T_) between A_T_ and D_T_ homoeologous gene copies for each of the commonly expressed genes were also visualized within the phylogenetic framework (Figure 4). The same relative tree topology between the accessions was observed in the gene expression tree and phylogenetic tree. The diploid F_1_-hybrid was relatively distant from natural tetraploids in the phylogeny and it had the least number of biased genes.

**Figure 5:**
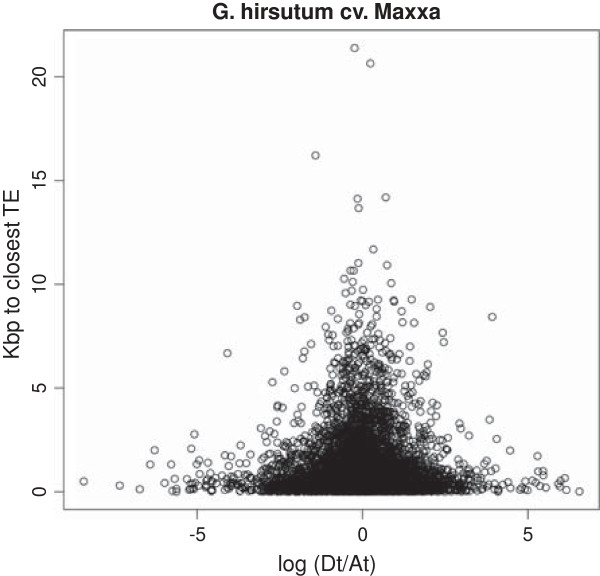
**A scatter-plot that relates gene expression and distance to the nearest TE of all commonly expressed genes (n = 11,469) in Maxxa.** Difference in fold-change between A_T_ and D_T_ (gene expression bias) is on the x-axis and the distance to the nearest TE is on the y-axis.

One explanation of biased gene expression could be the distance of a gene to the nearest transposable element. In other words, gene proximity to a TE quantitatively reduces the amount of gene expression due to heterchromomatic effects. For example, the greater-than-average A-genome bias of a subset of genes might be explained by greater distance between those genes and the nearest TE than the remainder of genes in the genome. In our data, TE proximity was only statistically associated with gene expression bias in Tx2094 (p = 0.04, Figure 5).

### Differential gene expression between the polyploids and diploids

**Figure 6:**
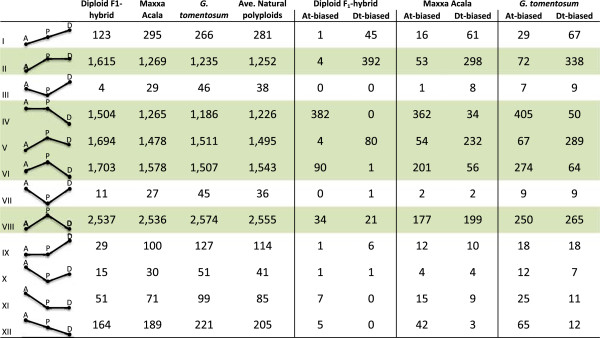
**Number of genes in 12 categories listed in first column where ‘A’ = expression from A genome, ‘D’ = expression level from D genome, and the ‘P’ = expression level from polyploid.** The position of letters A, D and P indicate the level of expression relative to the other. Rows shaded green indicate ‘high-level expression’ of the polyploid compared to the diploids.

Considering expression patterns for duplicated genes in a polyploid, relative to their orthologs in related diploids, twelve possible categories have been described, where each category corresponds to a combination of relative equalities or inequalities between two diploids and between the total expression in the tetraploid [18,19,27]. The expression level profile of each gene can be placed in one of these categories and their relative abundance has been used to determine expression level dominance (ELD, Figure 6, [4,18,19]). In this context, additive expression is when the tetraploid expression level is approximately the average of the diploid expression levels. Transgressive expression (upward or downward) refers to expression categories where the tetraploid expression is either higher or lower than that in both diploid parents. Expression level dominance categories are those in which the expression level in the tetraploid matches the expression level in one of the two diploids, whether expression in that diploid is higher or lower than in the other diploid (II, IV, IX, XI, Figure 6; [4]). In general, the 3 natural allotetraploids (Maxxa, Tx2094, and *G. tomentosum*) exhibited a consistent expression pattern (*i.e.*, approximately equal number of genes in equivalent categories). The diploid F_1_-hybrid frequently differed from the natural polyploids in gene expression levels and the categories of expression level dominance.

Counts of duplicated genes from this RNA-seq study were placed into the 12 expression levels categories and compared to the previously reported microarray results of petal transcriptomes (Additional file 4: Figure S4, [18]). Previously, a modest level D-genome ELD was found as a ratio between categories (Categories II and XI/Categories IV and IX). In our RNA-seq results, we did not find a discernable level expression level dominance because the ELD ratios were very close to 1 (0.92, 1.02, and 0.98 in the diploid F_1_-hybrid, Maxxa, and *G. tomentosum*, respectively). This disagreement between the microarray and RNA-seq results could have been due to an artifact of microarray probe design where more EST templates were available for the D-genome than the A-genome (*i.e.* floral ESTs were only obtained from the D-genome [46]). In addition, our use of SCAN UPC expression likelihoods reduced the amount of transcriptional noise by filtering the low-level expressed genes from the analysis (note the smaller total number of genes in each treatment, Additional file 4: Figure S4).

We found that the polyploid categories II and IV (greater expression of transcripts from both polyploid A_T_- and D_T_-genomes compared to diploids) are ~10-fold more abundant than categories IX and XI (less expression of transcripts from both polyploid A_T_- and D_T_-genomes compared to diploids; Figure 6). All categories where polyploid gene expression level was expressed as high (or higher) as the diploid with the highest expression level had many genes (generally >1,000; categories II, IV, V, VI, and VIII). All categories where polyploid gene expression level was expressed as low (or lower) as the diploid with the lowest expression level had very few genes (generally < 100; Categories III, VII, IX, X, and XI).

Because we could discern the A_T_ and D_T_ transcripts in our RNA-seq data, we were curious if the general trend of increased expression in the polyploid was due to an increase of transcription levels in a single genome or both genomes (Figure 6). Thus, we considered genes that had a significant expression bias within each of the 12 expression categories. For example, 1,504 genes exhibited expression level dominance of the A_T_-genome in the diploid F_1_-hybrid. Of those, 382 A_T_-D_T_ gene pairs contained homoeo-SNPs and had significant expression bias based on read counts of each homoeolog. All 382 of these gene pairs had a A_T_-genome bias and 0 genes had a D_T_-genome bias and while this was the most extreme difference, it represented a general trend within the expression categories. Generally, the direction of expression level dominance in all ‘increased expression’ categories (Categories II, IV, V, and VI) coincided with the RNA-seq read composition of genes with significant homoeolog expression bias. Perhaps, this also indicated *cis-*acting regulation of genes where a higher expression of one of the two diploid genomes was conserved in the polyploid nucleus and potential overexpression was controlled by and at the same levels as in the diploids.

Interestingly, category VIII is described as exceptional or transgressive expression in the polyploid (A = D < A_T_D_T_). It had equivalent contributions from each genome. In contrast to other categories, more genes were placed in this category on a percentage basis by our RNA-seq analysis than by the previous microarray experiment. There were also many fewer of these genes in the diploid F_1_-hybrid than in the natural polyploids. Perhaps this transgressive expression represent a different type of ‘additive’ gene expression where each diploid contributes an additive amount of transcript (such that two copies in the nucleus results in twice as much gene product), except that these loci have not individually evolved a mechanism for feedback control of expression. Such an interpretation would agree with a form of pseudo-overdominance (*i.e.* heterosis) between genomes and may have been due to complementary combinations of *cis-* and *trans-*acting factors resulting in more expression together than either genome alone.

### A simplified approach to comparisons of gene expression

We generated a new perspective of gene expression in polyploid cotton by cross-listing two separate gene lists. The first list consisted of the genes that were differentially expressed between the diploids, A_2_ and D_5_, and the second list consisted of the genes that had expression bias in each hybrid or polyploid. For this perspective, assume that the two diploid genomes actually contain the ancestral genomes of the extant polyploids and that the diploid F_1_-hybrid is simply an intermediate step in their evolution. Four groupings of genes are useful for this consideration: exhibiting no differential expression in diploids or polyploids, differential expression in diploids only, differential expression in polyploids only, or differential expression in both diploids and polyploids (Table 4). It was uncommon for genes that were not differentially expressed in the diploids to develop differential expression in the polyploids (113/6,358 in F1, average 943/6,358 in the natural polyploids). It was more likely for a differentially expressed gene in the diploid to become equally expressed in the polyploid than it was to stay differentially expressed (3,932 *vs.* 1,179 in diploid F_1_-hybrid, 3,476 *vs.* 1,635 in the natural polyploids). In genes that were differentially expressed in both diploids and polyploids, it was rare for the direction of bias to change from one direction in the diploids to the opposite direction in the polyploid (1% in diploid F_1_-hybrid, 16% in the natural polyploids). Inspection of 1,000 bp upstream of the annotated genes did not reveal any significant differences in the number of SNPs between these two categories of genes (*i.e.* same expression levels between diploid and polyploid *vs.* different expression levels in diploids and polyploids, data not shown). Perhaps the causative differences of expression were further upstream [47] or caused by an indel. When changes in expression bias direction were identified, the bias appeared to slightly shift more towards the A_T_-genome than the D_T_-genome. Such a simplified perspective overlooks some complexities of cotton polyploid formation; however, it emphasizes the putative predominance of *trans-*acting regulation of gene expression in polyploid cotton if the ‘different’ categories are generally interpreted as *cis-*acting regulation.

**Table 4:** **Number of genes with or without differential expression in the diploids and polyploids (for example, 6,358 genes were equally expressed in the diploids; of those, 6,245 were also found to be equally expressed in the diploid F**_**1**_**-hybrid and 113 were found to be differentially expressed in the diploid F**_**1**_**-hybrid)**

**Diploids**	** Equal**		** Different**		** Change**	
**Polyploids**	**Equal**	**Different**	**Equal**	**Different**	**A- > D**	**D- > A**
**Diploid F**_**1**_**-hybrid**	6,245	113	3,932	1,179	2	8
***G. tomentosum***	5,127	1,231	3,226	1,885	155	174
***G. hirsutum *** **maxxa**	5,509	849	3,566	1,545	102	125
***G. hirsutum *** **TX2094**	5,609	749	3,635	1,476	97	127

In addition, we compared the average degree of bias detected in the diploid F_1_-hybrid to the average degree of bias detected in the natural polyploids. When there was a significant D_T_ bias detected in the diploid F_1_-hybrid, there was also a significant difference in the amount of bias between the diploid F_1_-hybrid and the other polyploids. When there was a significant A_T_ bias detected in the diploid F_1_-hybrid, there was not a significant difference in the amount of bias between the F_1_ and the other polyploids.

To complement that previous perspective, we also compared the expression of the A_T_-genome to the diploid A-genome and the D_T_-genome to the D-genome (Table 5). The diploid F_1_-hybrid had fewer expression level differences with the diploids than did the natural polyploids (505 *vs.* 4,957, average). The A_T_-genome in the diploid F_1_-hybrid had more expression level differences with the diploid A-genome than the D_T_-genome did with the diploid D-genome (390 *vs.* 115), but the natural polyploids all had more expression level differences with the diploids in the D_T_-genome than in the A_T_-genome. When the polyploid had a different level of expression than its respective diploid, the A_T_-genome expression levels were more frequently higher than the A-genome diploid expression levels and the D_T_-expression levels were more frequently lower than the D-genome diploid expression levels.

**Table 5:** Changes in gene expression from diploid to tetraploid genomes

	**A**_**T **_**up**	**A**_**T **_**down**	**D**_**T **_**up**	**D**_**T **_**down**
**Diploid F**_**1**_**-hybrid**	235	155	47	68
***G. tomentosum***	1,396	1,294	1,624	1,719
***G. hirsutum *** **maxxa**	1,080	813	1,396	1,396
***G. hirsutum *** **TX2094**	1,060	880	1,084	1,129

“A_T_ up” means that A_T_ was more expressed than A_2_, etc.

## Discussion and conclusions

### Gossypium petal transcriptome

Cotton fiber tissue has been the main focus of many transcriptome studies of *Gossypium* species because of its economic importance [10-12,48,49]. In contrast, petal tissue is an excellent ‘model’ tissue for cotton gene expression because of its highly canalized development and limited interaction with the environment [18,20]. In one of the first applications of the *G. raimondii* reference genome [34], the gene expression levels in petals were determined by mapping RNA-seq reads to the annotated genes. Only 36-42% of the *Gossypium* genome was expressed in the petal tissue of open flowers prior to pollination. This number was noticeably smaller than the number of genes expressed during the development of cotton fiber [10], but we only sampled one time point of petal development. Between 75% and 83% of the expressed genes were expressed in every accession (Table 3). While these commonly expressed genes had a 99% probability of expression, they were not expressed at the same levels in the different accessions. Using gene expression as a metric [18], the expected phylogenetic relationships were clearly seen within a simple neighbor-joining tree containing two major clades (A and D, Figure 4).

Distributing transcripts in GO categories developed a molecular snap shot of the petal tissue. The cellular component ontology that includes multi-subunit enzymes and other protein complexes was most abundant GO category (88%). Petal cells undergo rapid elongation to reach full petal expansion stage. Actin cytoskeleton helps with cell elongation by transporting vesicles and organelles to the site of growth from cytoplasm. The cytoplasm (28%) and cytoplasmic parts (27%) were most represented under cellular component GO category. About 17% of transcripts fell under biological processes GO category and under this category cellular protein metabolic processes (31%) were most prominent. Petal tissue is an energy sink tissue for plant reproduction where starch and sucrose are mobilized from photosynthetic organs and broken down to sugars that function as precursors to essential primary and secondary metabolites [50]. This was supported by the transcript abundance of different KEGG pathways. Many enzymes expressed in petal tissue were involved in starch and sucrose metabolism pathways.

In addition to a comprehensive assessment of gene expression in petals, the improvements in our analytical methodology resulted in three refinements of our understanding of the cotton transcriptome. First, both Yoo et al. [19] and this study found that expression bias is only found in a minority of genes. While previous studies used EST assemblies, we used an assembled genome reference and its corresponding gene annotations to determine gene expression resulting in more accurate numbers of expressed genes in a single tissue and the individual contributions of the distinct polyploid genomes.

Second, UPC was used to assign an expression probability to each gene. Genes with low expression levels have less accuracy and cause inflated comparisons of expression level differences due to overlapping standard deviations of expression (*e.g.,* A is equal to P; D is equal to P, but A is not equal to D). Elimination of low-level expressed genes emphasized two clear trends of the polyploid transcriptome. The first trend was that we found negligible ‘down-regulation’ in the polyploid. When the low-level expressed genes (instead of a fold-change threshold) were eliminated, the frequency of categories of ‘down-regulation’ in the polyploid (III, IX, X, XI) were negligible compared to the frequency of ‘up-regulation’ categories (II, IV, V, VI). The second trend was that we found no ELD in the cotton petal transcriptome. Previously, a small D-genome ELD was found in petals, but it could be potentially explained by the origin of the microarray probes (Additional file 4: Figure S4). A small A-genome ELD in cotton leaves [19] could partially be explained by the difference of evolutionary distance between A_T_ and A_2_, and D_T_ and D_5_. Since A_T_ and A_2_ are ~2× closer in sequence similarity than D_T_ and D_5_, the A_T_-genome reads of the polyploid are more likely to map as equally well (or equally poor) as the A-genome diploid than the D-genome sequences, particularly when an EST assembly of A_T_ and D_T_ is used a reference. This potential inherent bias is fully considered when SNP-tolerant mapping is used since SNP positions are masked during the read alignment resulting in normalized read mapping efficiencies. This study was the first study to use SNP-tolerant mapping in RNA-seq analysis of a polyploid transcriptome.

Third, when the polyploid had equal to the higher diploid parent (*i.e.* up-regulation categories II, IV, V, VI), it was previously found that the expression level of the polyploid was explained by an increased expression of the ‘recessive’ parent [19]. We found no evidence for a significant contribution from the ‘recessive’ parent. In fact, we observed an exceptional amount of expression stability between the diploid and polyploid petals. This paradoxical finding could be due to the differences between leaf and petal, or it could be due to the analytical refinements mentioned above. Further examination of additional RNA-seq dataset will contribute a greater understanding of ELD.

### Evolution of cotton gene expression

Our results contribute to a growing understanding of the evolution of gene expression in cotton. Adams *et al.,*[8] first reported expression bias in natural *Gossypium* polyploids. Since each gene and its corresponding *cis-*acting regulatory sequence(s) of the diploid A- and D-genomes can be traced to a common ancestry 5–10 mya [1], the cause of differential expression between homoeologs remained a mystery. Using microarray technology, homoeologous expression biases were observed in petal tissues from five *Gossypium* polyploids and a diploid F_1_-hybrid [18,20]. These previous observations suggested two phases or modes for the evolution of cotton gene expression during polyploidization. Similar to Yoo et al. [19], our results suggest three phases of evolution that ultimately determine the expression levels of duplicated genes in the cotton genome.

The first phase consists of independent changes in upstream, *cis-*acting regulatory regions of genes prior to genome hybridization. In cotton, speciation of the two diploid genomes followed distinct evolutionary trajectories as evidenced by their differential accumulation of retrotransposons [51]. During speciation, the *cis-*acting promoter regions of protein coding genes also likely changed for a modest number of genes. Evidence of changes to *cis-*acting regulatory regions was observed by the conservation of homoeologous gene expression bias. If the observed expression biases were the result of a stochastic process during polyploid formation, then the direction of homoeologous expression bias would also be stochastic. When we inspected *all* genes with an expression bias in more than one accession (Figure 3A), we found that direction of bias was nearly always conserved in the four different accessions and in two different hybridization events (e.g. Figure 3B). Thus, the direction of expression bias of these loci may have been due to differential *cis-*acting regulation efficiency that evolved in the diploids and was subsequently conserved or maintained in the polyploid nucleus. This manner and level of conserved *cis*-acting regulation has not been previously reported. Previously, a mode of gene regulation (*i.e. cis-*) could only be ascribed to the small number of genes where the polyploid expression patterns were different that the diploids (Table 5).

Alternatively, or in addition, to *cis-*acting modification, transposable elements could have region of influence causing a quantitative reduction of transcription factor binding and correspondingly a reduction of transcription initiation [52]. Indeed, TEs have been differentially amplified and inserted in the A- and D-genomes [53]. A correlation of gene distance to the nearest TE in the D-genome reference sequence was only significant in one of the five datasets we tested. Thus, there was not a strong association between TE distance and expression bias, even with a ~2× difference in genome size between the A- and D-genomes largely due to TE amplification and re-insertion. While a significant difference between categories of genes (A-biased or D-biased) was not apparent, only the distances from a single annotated reference genome were used (D-genome). We anticipate that a more meaningful comparison will be possible once the A-genome sequence has been published. A publically accessible A-genome sequence would allow the actual distances between A-genome TE insertions and annotated genes to be used with the current D-genome distances for a more biologically meaningful comparison.

The second phase of duplicate gene evolution is hybridization. When two genomes are combined into a single nucleus, they share gene regulatory factors, which can lead to novel regulatory processes and changes in regulatory networks. These novel interactions have been considered as a genome shock that accompanies polyploidization and may provide a new source of genetic variation [29], see [45] for recent review. However, we found that most homoeologous genes were not expressed at statistically different levels in petals from tetraploids. If *trans-*acting factors were regulating homoeologous gene expression, both homoeologous loci would be expressed at similar or equal levels (*e.g.* Table 5). Our results suggest that either the expression levels of most genes are controlled to some degree by *trans-*acting factors or that our experimental design lacked sufficient power to detect a significant difference between homoeologous expression levels. With only three replicates, we had modest empirical estimates of power in our dataset. While some real homoeologous differences remained undetected, the general interpretation of the relative amounts of *cis-* and *trans-*acting regulation will be evaluated against future studies. Previous reports of an overall genome bias (D_T_ > A_T_ composed of many genes with small differences) due to either accelerated evolution of one of the two genomes or a mechanism of epigenetic control of expression, did not agree with our current findings [18,20]. Perhaps, the previous use of DNA microarrays resulted in an ascertainment bias through probe construction.

The second phase of gene evolution can also be interpreted through the lens of expression level dominance [4,18,27]. Nucleolar dominance is one type of molecular interaction that occurs when two genomes merge to form a polyploid and it is mediated by the targeting of specific siRNAs [54,55]. Expression level dominance is a more generalized phenomenon (without a sequence-specific trigger) and it was found in the diploid F_1_-hybrid and natural *Gossypium* allopolyploids in previous microarray-based and RNA-seq studies [18,19,27]. We did not detect a consistent pattern of expression level dominance in our analysis. Within the comparative categories of gene expression that identify expression level dominance, the polyploid expression levels more commonly matched or exceeded the diploid species with the greater level of expression, independent of whether that diploid had the A- or D-genome. This consistent polyploid ‘high-level expression’ appeared to be a general trend that could also be explained by *cis-*acting transcriptional regulators. For example, suppose that the A- or D-genome has a *cis-*acting regulating sequence that is more efficient than the other genome’s homoeologous sequence due to mutation during the first phase of evolution. When combined into a polyploid nucleus, a more efficient *cis-*acting regulator would continue to induce one of the two homoeologous genes at the requisite level for expression in the polyploid nucleus just as it had in the diploid nucleus (*i.e.* polyploid expression was the same as the higher expressing diploid). The less common events where the polyploid had lower expression levels could be due to *trans-*acting transcription repressors where the active repressor of one genome reduces expression levels in both genomes.

In the second evolutionary phase, we can also consider the constitution of the polyploid expression levels in the categories that match or exceed the expression levels of the diploids because we can distinguish between A_T_ and D_T_ transcripts. For example, if the polyploid matched or exceeded the expression level of the A-genome (Category IV and VI), the polyploid transcripts would largely consist of A_T_ transcripts and not D_T_ transcripts. The reverse was true for categories II and V where the polyploid matched the expression level of the D-genome diploid. Furthermore, most (50-70%) of the genes in the ‘expression level dominance’ categories actually showed no change in the A_T_- relative to the A-genome and in D_T_- relative to D-genome suggesting similar controls of expression between the diploid and the respective polyploid.

The third phase of gene expression evolution occurs between hybridization and adaptation to the species current biological niche. We found a larger number of biased homoeologous genes in the natural polyploids when compared to the diploid F_1_-hybrid in agreement with Flagel et al. [18]. Since we had relatively similar amounts of statistical power for each accession, the increased number of genes detected with a homoeologous bias could have evolved since formation of the ancestral polyploid. When a significant bias was detected between the homoeologs, the difference was greater in the D_T_ genomes of the natural polyploids than it was in the genome of the diploid F_1_-hybrid (but not the A_T_ biased genes). Perhaps, this observation represents a higher rate of mutation accumulation in the A_T_-genome *cis-*acting regulatory regions than the homoeologous D_T_-genome regions. It also agreed with our previous finding of general nucleotide diversity between the two genomes [36], our general findings of overall expression in Table 4, and previous reports [19]. Although rate heterogeneity seems unlikely, a relative rate test cannot be evaluated without the genome sequence of an appropriate outgroup (*e.g. G. kirkii*).

Here we used RNA-seq to characterize gene expression in polyploid cotton petals with an unprecedented level of resolution within each genome. Future comparisons of these findings to similar results in other polyploids will perhaps enable an extrapolation of general trends to unifying concepts of polyploid gene expression in plants. Collectively, these results suggest that after polyploidization 1) most homoeologous gene pairs are expressed at approximately equal amounts, 2) ~20% of expressed genes have biased expression, 3) a portion of these genes have expression biases in more than one polyploid and the direction of their bias is largely conserved, 4) for any set of genes with expression biases in more than one accession, only a very a small number of genes have a direction of expression bias that was unique or contrary to the observed direction in other accessions, and 5) that general trends of gene expression can be interpreted as *cis-* and *trans-*acting regulation of polyploid gene expression. We recognize that the simple explanations of increased and decreased levels of expression genes represent only a few of many possible combinations of *cis-* and *trans-*acting factors on gene expression within discrete and interconnected biochemical pathways. Indeed, the modest number of changes to polyploid gene expression corresponded to the limited number of genomic changes that were previously found to accompany polyploidization in cotton [56]. Perhaps, these exceptional gene expression biases indicated an additional level of gene expression regulation such as DNA methylation. The interesting exceptions to a conserved direction of gene expression bias and their potential effects on the phenotype merit further investigation.

## Authors’ contributions

AR handled the plant material, prepared the libraries for sequencing, and helped write the manuscript. JTP performed the computational and statistical analyses and helped write the manuscript. JAU performed additional summaries of the results and wrote the manuscript. All authors read and approved the final manuscript.

## Supplementary Material

Additional file 1: Figure S1 Distribution of gene ontology (GO) terms in 11,469 commonly expressed petal genes. a) Biological process; b) Cellular component; and c) Molecular function. Click here for file

Additional file 2: Figure S2 The enzymatic pathways can be divided into four general KEGG pathways: Metabolic pathways, Biosynthetic pathways, Degradation pathways and signaling pathways. Click here for file

Additional file 3: Figure S3 Differences in log base 2 RPKM between the A_T_ and D_T_ genomes of each cotton species. Each histogram shows the number of genes (of the 11,469 commonly expressed petal genes) with each magnitude of homoeologous bias for Maxxa, G. tomentosum, the F_1_ diploid hybrid, or T×2094. Positive values indicate an At bias, while negative values indicate a Dt bias. Click here for file

Additional file 4: Figure S4 Counts of duplicated genes from this RNA-seq study were placed into the 12 expression levels categories (identical to Figure 6), but here we have included the numbers from previously reported microarray results of petal transcriptomes [18] for comparison. Click here for file
