# Supplementary figures and images for: Polyploidy and the petal transcriptome of Gossypium
Source: BMC Plant Biol. 2014 Jan 6;14:3. doi: 10.1186/1471-2229-14-3 (PMC3890615; doi:10.1186/1471-2229-14-3)

## Slide 1
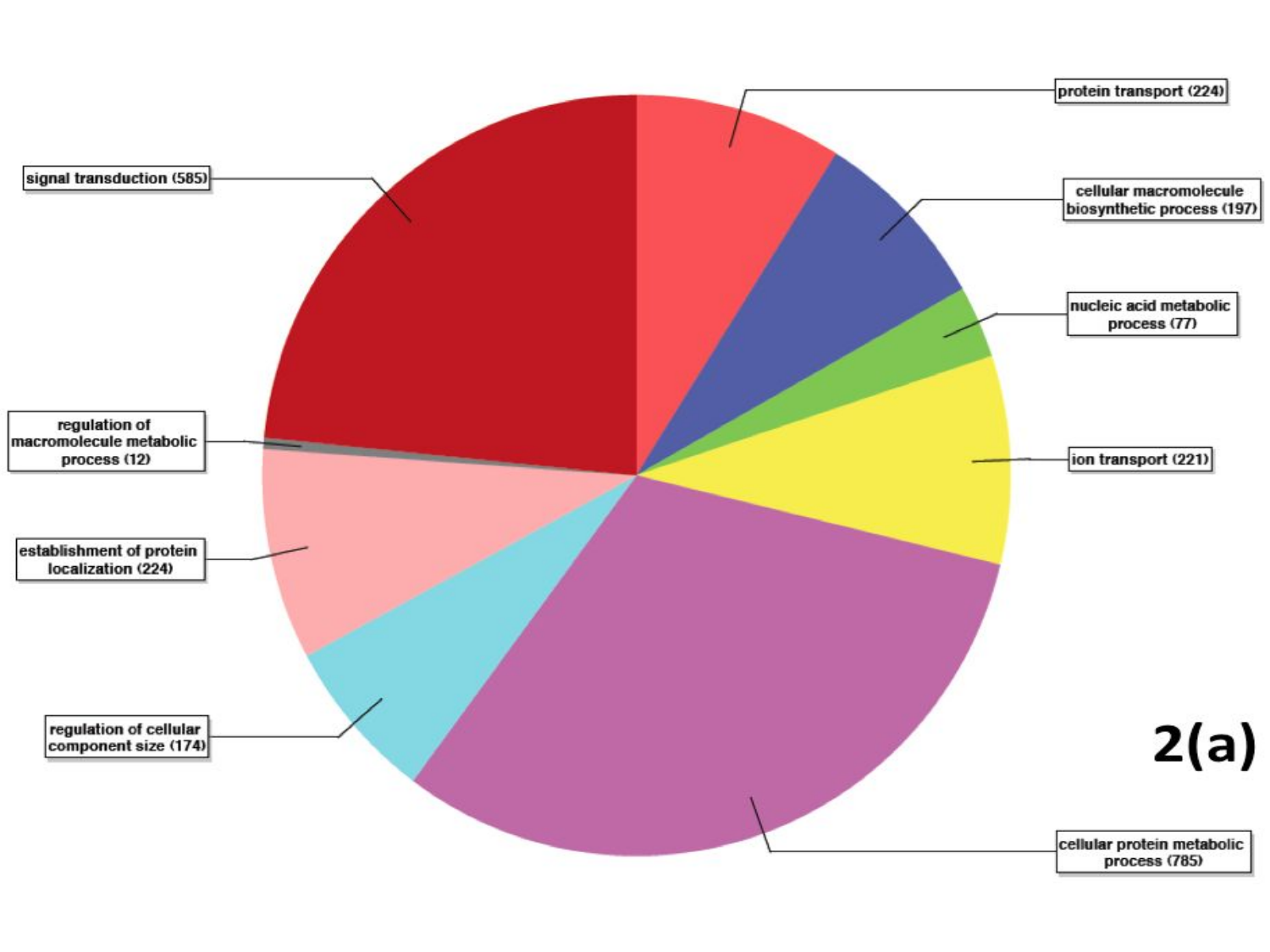

## Slide 2
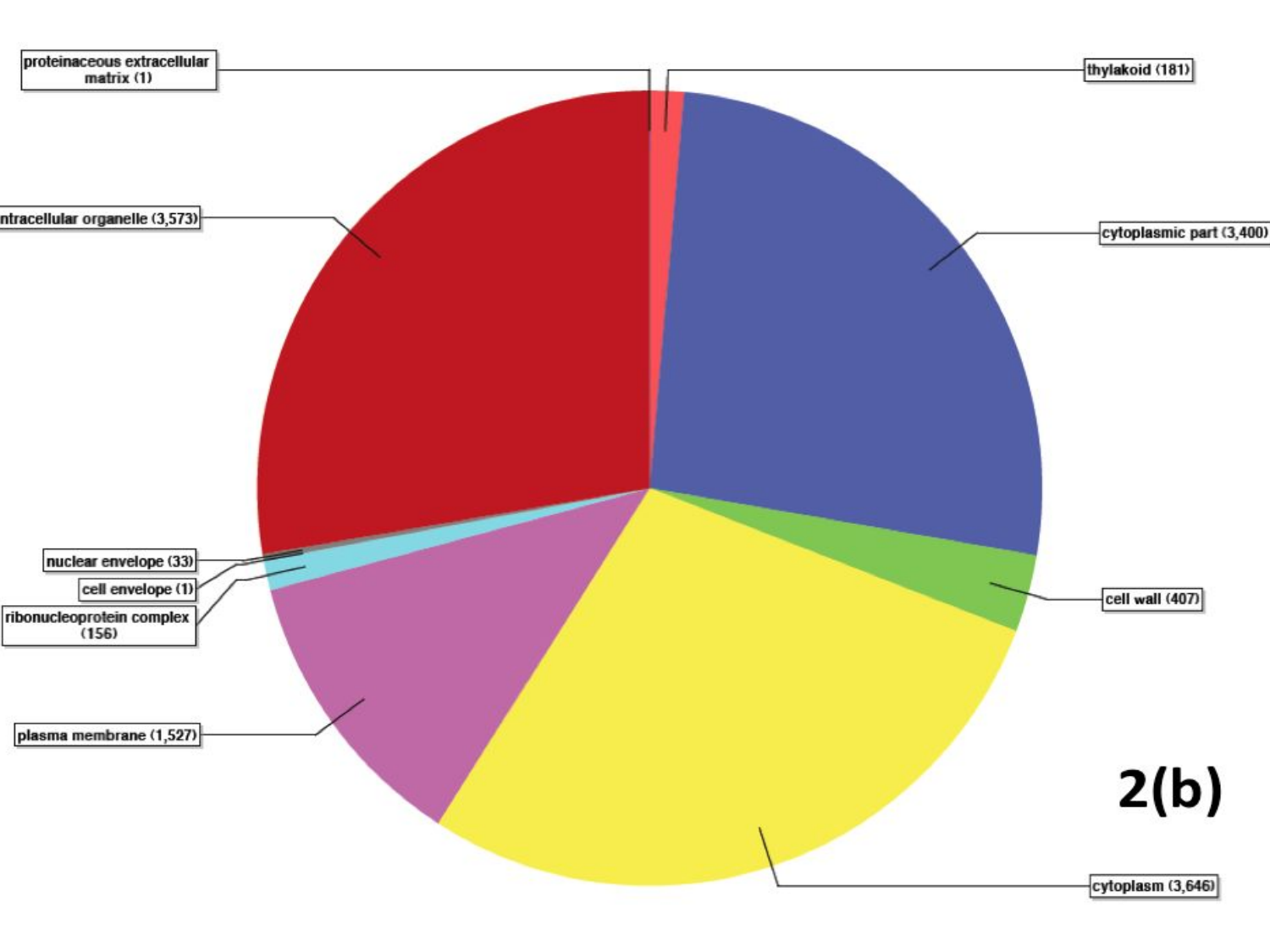

#

## Slide 3
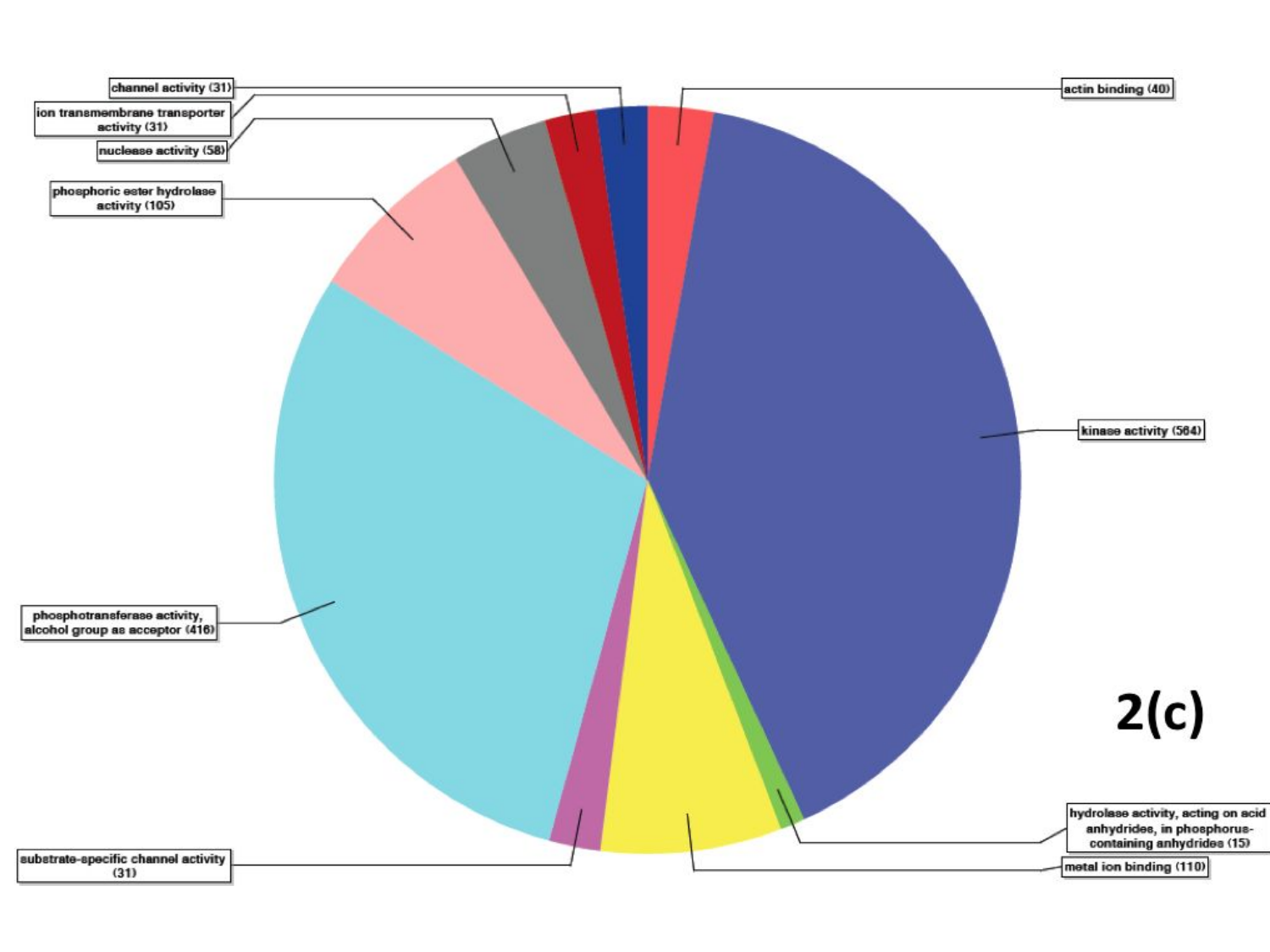

#

Supplement: Additional file 1: Figure S1 — Distribution of gene ontology (GO) terms in 11,469 commonly expressed petal genes. a) Biological process; b) Cellular component; and c) Molecular function. [file 1471-2229-14-3-S1.pptx]

# KEGG pathway representation

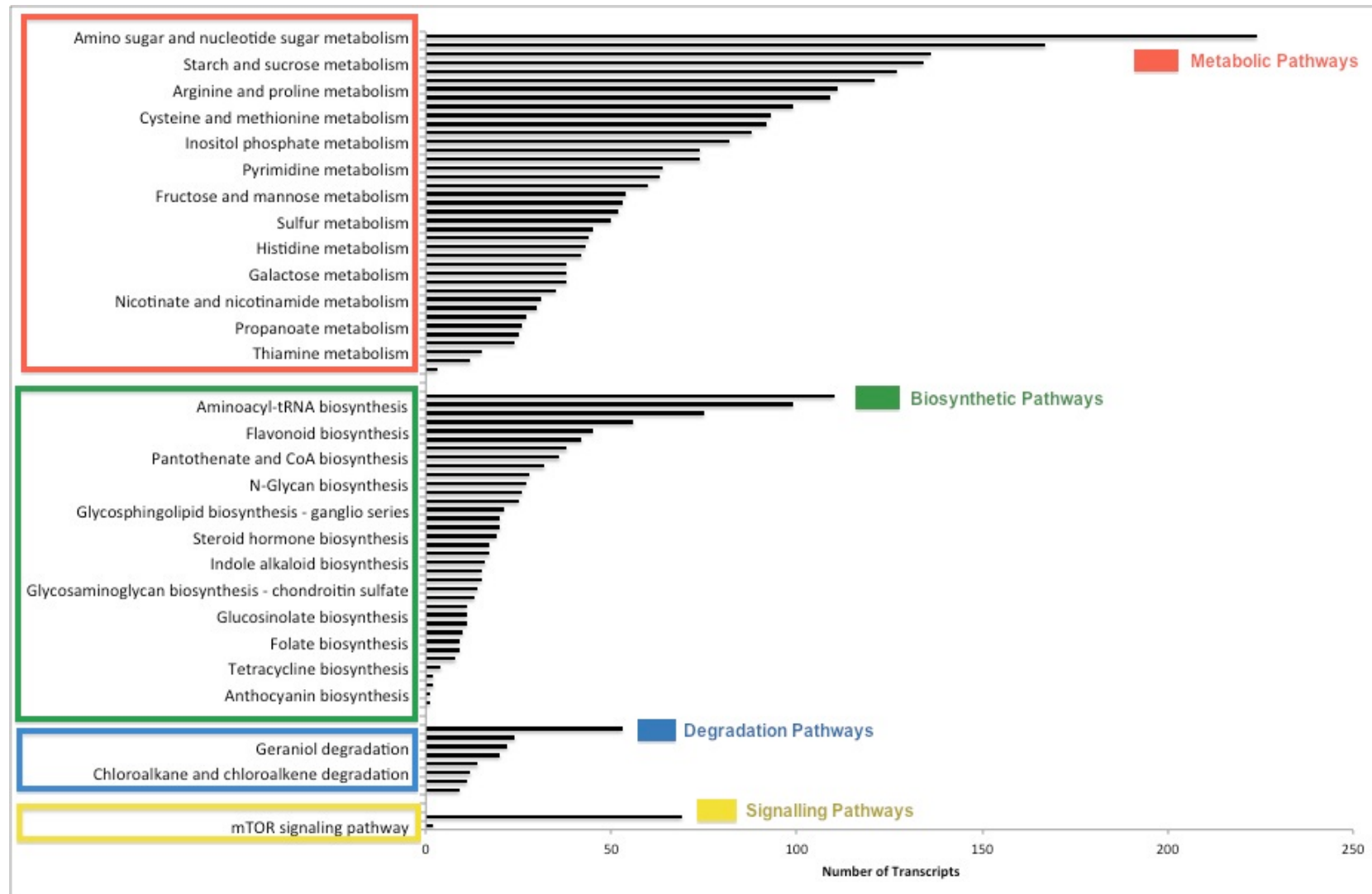

Supplement: Additional file 2: Figure S2 — The enzymatic pathways can be divided into four general KEGG pathways: Metabolic pathways, Biosynthetic pathways, Degradation pathways and signaling pathways. [file 1471-2229-14-3-S2.pdf]

## Slide 1
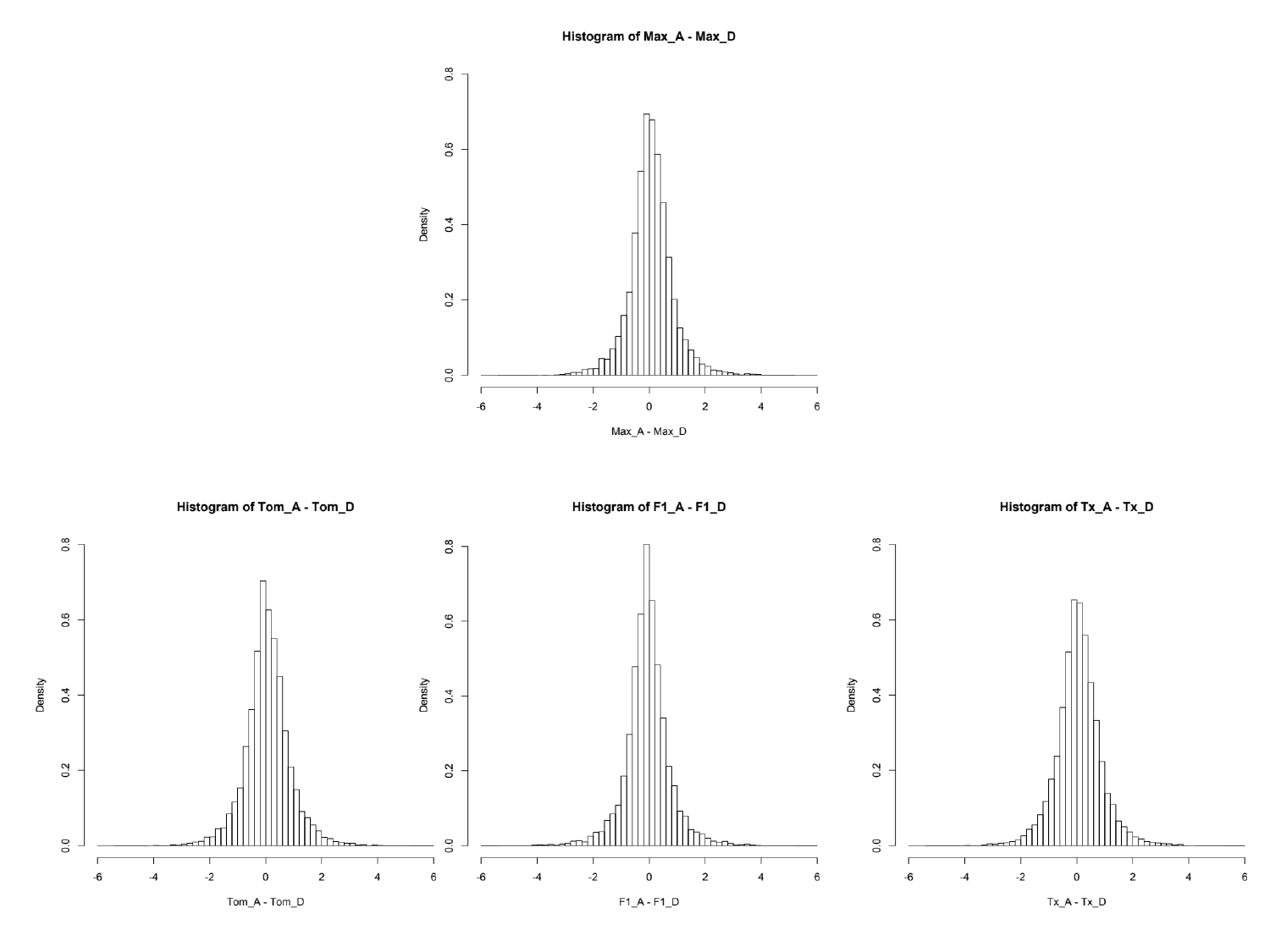

Supplement: Additional file 3: Figure S3 — Differences in log base 2 RPKM between the AT and DT genomes of each cotton species. Each histogram shows the number of genes (of the 11,469 commonly expressed petal genes) with each magnitude of homoeologous bias for Maxxa, G. tomentosum, the F1 diploid hybrid, or T×2094. Positive values indicate an At bias, while negative values indicate a Dt bias. [file 1471-2229-14-3-S3.pptx]
