# Supplementary material for: Polyploidy and the petal transcriptome of Gossypium
Source: BMC Plant Biol. 2014 Jan 6;14:3. doi: 10.1186/1471-2229-14-3 (PMC3890615; doi:10.1186/1471-2229-14-3)
Supplement: Additional file 4: Figure S4 — Counts of duplicated genes from this RNA-seq study were placed into the 12 expression levels categories (identical to Figure 6), but here we have included the numbers from previously reported microarray results of petal transcriptomes [18] for comparison. [file 1471-2229-14-3-S4.pdf]

		Diploid F1-hybrid		Maxxa		*G. tomentosum*		Ave. Natural polyploids		F1		Mx		Tom	
		RNA-seq	Microarray	RNA-seq	Microarray	RNA-seq	Microarray	RNA-seq	Microarray	At-biased	Dt-biased	At-biased	Dt-biased	At-biased	Dt-biased
I	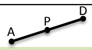	123	1,581	295	1,581	266	2,064	281	1,823	1	45	16	61	29	67
II	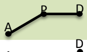	1,615	4,888	1,269	3,240	1,235	2,927	1,252	3,084	4	392	53	298	72	338
III	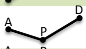	4	248	29	621	46	1,489	38	1,055	0	0	1	8	7	9
IV	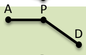	1,504	2,264	1,265	3,496	1,186	3,578	1,226	3,537	382	0	362	34	405	50
V	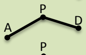	1,694	69	1,478	553	1,511	1,030	1,495	792	4	80	54	232	67	289
VI	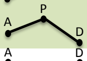	1,703	168	1,578	523	1,507	1,290	1,543	907	90	1	201	56	274	64
VII	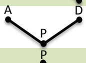	11	452	27	1,855	45	4,387	36	3,121	0	1	2	2	9	9
VIII	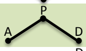	2,537	302	2,536	1,563	2,574	3,666	2,555	2,615	34	21	177	199	250	265
IX	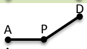	29	1,951	100	4,444	127	3,857	114	4,151	1	6	12	10	18	18
X	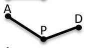	15	60	30	497	51	1,036	41	767	1	1	4	4	12	7
XI	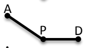	51	4,629	71	4,016	99	3,403	85	3,710	7	0	15	9	25	11
XII	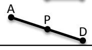	164	1,951	189	1,747	221	2,176	205	1,962	5	0	42	3	65	12
